# Development and characterization of the genotype F attenuated mumps candidate strains

**DOI:** 10.3389/fimmu.2025.1629585

**Published:** 2025-07-22

**Authors:** Hongtao Liu, Na Liu, Yueqiu Sun, Shuang Li, Hang Li, Menghan Wang, Hui Shuang, Yan Cai

**Affiliations:** ^1^ Vaccines R&D Department, Changchun Keygen Biological Products Co., Ltd., Changchun, Jilin, China; ^2^ Vaccines R&D Department, Changchun Institute of Biological Products Co., Ltd., Changchun, Jilin, China; ^3^ State Key Laboratory of Novel Vaccines for Emerging Infectious Diseases, China National Biotec Group Company Limited, Beijing, China

**Keywords:** mumps virus, F genotype, QBB strain, immunogenicity, neurovirulence

## Abstract

**Background:**

Mumps is an acute infectious disease caused by the mumps virus (MuV), primarily affecting the parotid glands, though it can also lead to systemic infections, including the nervous system. In China, the predominant circulating MuV genotype is F, while the vaccine strain (S79 or WM84) belongs to genotype A, raising concerns about immunization effectiveness.

**Methods:**

A genotype F MuV strain was isolated from throat swabs of six suspected mumps patients. Through cell adaptation passage and plaque purification, two candidate vaccine strains QBB-2BS-3.2 and QBB-2BS-9.3 were prepared. Their immunogenicity was assessed by neutralizing antibody and cell-mediated immune responses in immunized mice. Additionally, neurotoxicity was evaluated in neonatal Lewis rats.

**Results:**

Both QBB-2BS-3.2 and QBB-2BS-9.3 elicited strong neutralizing antibody responses and robust cell-mediated immune responses in mice. Notably, neurotoxicity testing revealed minimal neurotoxicity in QBB-2BS-3.2 and QBB-2BS-9.3 strains, comparable to the S79 vaccine strain.

**Conclusions:**

This study successfully developed two attenuated genotype F MuV candidate strains with favorable immunogenicity and safety profiles, laying a critical foundation for the development of genotype F mumps live attenuated vaccines.

## 1 Introduction

Mumps is an acute, self-limiting respiratory infection caused by the mumps virus (MuV), with the main clinical symptoms being fever, non-purulent swelling, and pain in the parotid glands (1, 2), which may also cause multi-organ damage, leading to serious complications such as pancreatitis, orchitis, deafness, aseptic meningitis, and encephalitis (3, 4). MuV belongs to the *Paramyxoviridae* family (*Paramyxoviridae*), whose viral genome is a non-segmented, single-stranded, negative-stranded RNA containing 15,384 nucleotides, encoding the transcriptional units of seven viral proteins, including nucleoprotein (N), phosphoprotein (P), matrix (M), fusion (F), small hydrophobic (SH), hemagglutinin-neuraminidase(HN), and large (L) proteins (5, 6). HN protein is a type II integral membrane protein. F and HN proteins work together on viral membrane proteins, play an important role in the binding and entry process of viruses into host cells, and are also the main antigens that elicit an immune response from the organism (7–9). The immunogenicity of the HN protein is closely associated with the spatial conformation of its surface-exposed antigenic epitopes. Studies indicate that three key regions (aa265–288, aa329–340, and aa352–360) on the HN protein surface exhibit strong antigenicity due to their exposure on the molecular surface. Among these, the aa329–340 region induces potent neutralizing antibodies in mouse models, confirming its role as a critical conformational epitope (9). Furthermore, experiments with the HN3 fragment (aa213–372) validate that this region simultaneously elicits both hemagglutination-inhibiting and neutralizing antibodies, highlighting its function as a core target for humoral immunity (10). Mumps virus consists of only one serotype, and the SH gene is highly variable and is often used as the basis for mumps virus genotyping. Based on the nucleotide sequence differences in the SH gene, mumps viruses are classified into 12 genotypes, including A, B, C, D, F, G, H, I, J, K, L, and N (11).

Vaccination is an effective means of preventing and controlling the spread of mumps (12). Since 1967, with the widespread use of live attenuated vaccine (LAV), especially the measles, mumps, and rubella vaccine (MMR), which has been incorporated into the national vaccination programs of many countries, the incidence of mumps has been significantly reduced. The most widely used live attenuated mumps vaccine is the Jeryl Lynn strain (genotype A), and its derivatives, such as RIT4385, S79, and Wm84, are also widely used. The live attenuated mumps vaccine strains mainly used in China are S79 and Wm84. Epidemiological data showed that the predominant genotype of MuV prevalent in China is the F genotype (13–15). MuV has only one serotype, and antigenic cross-protection exists between genotypes. Studies reported that cross-protection between genotypes is limited. Serum antibodies from the Jeryl Lynn strain could neutralize other genotypes of MuVs. However, the neutralizing antibody titers against other strains were significantly lower than its own (16, 17). It implied that the current genotype A vaccine may not provide sufficient protection against an epidemic of genotype F MuV, which may be the reason for the breakthrough mumps cases that have occurred even after vaccination (18–20).

Currently, the live attenuated mumps vaccine is mainly produced using primary chicken embryo cells (21, 22). A large number of chicken embryos were consumed, contrary to the 3R principles (Replacement, Reduction, and Optimization) advocated by the World Health Organization (WHO) for the use of animals (23). Moreover, chicken embryo cells as a heterologous cellular matrix may cause allergic reactions, and chicken embryo cells carry infectious avian retroviruses that can cause side effects (24–26). To improve the scale-up production efficiency of mumps-associated vaccine and improve product safety, there is an urgent need to develop new MuV-adapted cell lines to replace the traditional chicken embryo cell culture.

In this study, we successfully isolated one positive sample “MuV-QBB”, which was identified as genotype F MuV by isolating clinical samples from throat swabs of mumps patients. Subsequently, several F-genotype MuV 2BS cell-adapted strains were prepared using passaging and plaque purification. Finally, the candidate strains were tested for genetic stability, immunogenicity, and neurotoxicity. These results provided a basis for the development of the F genotype live attenuated mumps vaccine and the prevention and control of mumps.

## 2 Materials and methods

### 2.1 Animals and ethics statement

The SPF female BALB/c mice (6–8 weeks old) were purchased from the Changchun Institute of Biological Products Co., Ltd. (Changchun, China), the SPF pregnant female Lewis rats were purchased from Beijing Vital River Laboratory Animal Technology Co., Ltd.(Beijing, China), which were maintained under SPF conditions with a 12 h/12 h light/dark cycle. All animal experiments were carried out under the guidelines of the Council on Animal Care and Use, with protocols approved by the Animal Ethics Committee of the Changchun Institute of Biological Products. Animals were monitored daily and received free access to water and food throughout the study. Mice were euthanized by cervical dislocation.

The only human materials used were collected from the clinical suspected mumps patients for the purpose of public health and disease control. The studies involving humans were approved by the Ethics Committee of the China-Japan Union Hospital of Jilin University. The studies were conducted in accordance with the local legislation and institutional requirements. Written informed consent for participation in this study was provided by the participants’ legal guardians/next of kin.

### 2.2 Mumps clinic specimens collection and virus isolation

Six throat swab samples of children with mumps from an epidemic in Changchun City, Jilin Province, were collected and preserved after filtration and sterilization using a 0.22-micron filter. Within a biosafety level 2 (BSL-2) facility, clinical mumps specimens prescreened positive by reverse transcription polymerase chain reaction (RT-PCR) were inoculated onto confluent Vero cell monolayers in slant tubes for viral adsorption. Post-adsorption, the inoculum was replaced with maintenance medium. Cultures were incubated at 35°C with daily monitoring for cytopathic effect (CPE). Viral harvest occurred when CPE exhibited >90% cellular degeneration or upon completion of 7-day incubation. The strain was purified and separated using limiting dilution in Vero cells by picking single-plaque with the double-layer agar method. And the resulting isolation was stored at 4°C. After two rounds of plaque purification, cultures that appeared CPE continued to be inoculated with Vero cells for viral passaging.

### 2.3 50% cell culture infective dose assay

Virus titration was carried out using the CCID_50_ assay on Vero cells. The assay was performed in 96-well plates. Briefly, 10-fold serial dilutions (10^-1^-10^-7^) of viral samples were prepared in MEM (Gibco, 11095080) containing 2% FBS (RUNSUN, 200420100). Then, 100 μL of each virus dilution and 100 μL Vero cell suspension (1.6 ×10^5^ cells/mL) were added to each well. Then, the plates were incubated at 37°C in 5% CO_2_ for 7 days. After the incubation period, the cells were observed for cytopathic changes. The titer was calculated as the CCID_50_/mL using the Reed-Muench method.

### 2.4 Western blotting assay

Cells cultured in 6-well plates were lysed using RIPA lysis buffer (Beyotime, P0013B). Samples were separated by SDS-PAGE (Beyotime, P0052B) and transferred to a nitrocellulose membrane. The membrane was blocked with 6% non-fat milk (Solarbio, D8340) for 1 h and incubated at 4°C overnight with appropriate primary antibodies: anti-HN (Detai, DT7988-1) and anti-NP (Eastcoast, HM400). Then, the membrane was washed and incubated with HRP-labeled secondary antibody (Abcam, ab205719) at room temperature for 1 h for subsequent detection using enhanced chemiluminescence.

### 2.5 TEM

TEM assays were performed as described. Briefly, viral particles were adsorbed onto glow-discharged carbon-coated copper grids (300 mesh) by incubating 7 μL of the sample suspension for 3 min at room temperature. Grids were treated with 2% phosphotungstic acid for 2 min (Solarbio, G1870). Stained grids were air-dried in a desiccator overnight to preserve structural integrity. All steps were performed using fresh staining solutions to minimize artifacts. Electron photomicrographs were taken from virus structures under a transmission electron microscope (Hitachi, HT7800).

### 2.6 Sequencing and phylogenetic analyses

Viral RNA was extracted from culture with cytopathic effect by using the TaKaRa MiniBEST Viral RNA/DNA Extraction Kit (TaKaRa, 9766). To obtain the complete SH gene sequences, RT-PCR was performed with the One-step RT-PCR kit (TaKaRa, RR096A) following the manufacturer’s instructions. The primers of SH-1(5′-AATATCAAGTAGTGTCGATGA-3′) and SH-2 (5′-AGGTGCAAAGGTGGCATTGTC-3′) were used to amplify the entire SH gene. After purification of the PCR products with a QIA Gel Extraction Kit (Qiagen, 28704), the sequences were determined using the Sanger dideoxy terminator sequencing method with a BigDye Terminator Version 3.1 Cycle Sequencing kit (Life Technologies, 4337455) and ABI PRISMTM 3100 Genetic Analyzer (Life Technologies, Japan). Sequencher software version 5.0 (Gene Codes Corporation) was used to edit and assemble the raw sequence data to obtain the 316-nt complete SH gene sequences (2). Each dataset was used to build a neighbor joining phylogenetic tree with Mega5 using the maximum composite likelihood nucleotide substitution model. The topology of the phylogenetic tree was tested with 1000 bootstrap replications. Bootstrap values greater than 80% were indicated on the trees. Maximum likelihood tree was also generated with Mega. p-distances were computed in Mega. The number of synonymous nucleotide substitutions per synonymous site (dS) and the number of nonsynonymous substitutions per nonsynonymous site (dN) were estimated in Mega by Nei and Gojobori’s method.

### 2.7 Vaccine immunization program

Female BALB/c mice aged 4–6 weeks were divided into three groups, the experimental group, the positive control, and the negative control. The experimental group (the QBB-Vero group, QBB-2BS-3.2 group, QBB-2BS-3.3 group, QBB-2BS-3.4 group, QBB-2BS-9.3 group) received an subcutaneous (SC) injection of 100 μL mumps virus at 1 × 10^6^ CCID_50_/mL. The virus was administered via two SC injections (a primer and a booster after a two-week interval). Using the same vaccination schedule, the positive and negative control groups were administered with live MuV (S79) and PBS, respectively. Blood and spleen samples were obtained at 2 weeks post the booster administration.

### 2.8 Enzyme-linked immunosorbent assay

MuV-specific total IgG antibody titers were determined using ELISA. 50 μL of MuV mixtures (1 × 10^5^ CCID_50_/mL of each genotype A and F) was added to each well and coated overnight in 96-well plates at 4°C, and plates were washed three times with PBST (0.05% v/v) and blocked with 1% BSA (Beyotime, ST023) in PBST for 2 h at 37°C. Mouse serum was serially diluted and incubated with these mixtures for 1 h at 37°C, following which, the mixtures were washed, probed with HRP-conjugated goat anti-mouse IgG secondary antibodies (1:2000) for 1 h at 37°C, and washed again with PBS/T (0.05% v/v). Tetramethylbenzidine (TMB) (Thermo, XB3498751) was then added as a substrate, and the reaction was quenched by adding the stop solution for TMB. For each sample, the optical density (OD) was measured at 450 nm using an ELISA reader and correlated to values in the standard curve. MuV-specific total IgG antibody titers were tested in 3 wells per mouse, and the mean values were used for statistical analysis.

### 2.9 Mumps virus neutralization assay

The neutralizing antibody titers against the MuVs were determined using a microneutralization assay based on cytopathic effect (CPE) reduction. Briefly, heat-inactivated serum samples (56°C for 30 min) were serially diluted twofold in Modified Eagle Medium (MEM) supplemented with 2% fetal bovine serum (FBS), starting from an initial dilution of 1:4. Each serum dilution was mixed with an equal volume of MuVs strain (QBB or S79 MuVs, 500–2000 CCID_50_/mL) and incubated at 37°C for 1 h for neutralization. The virus-serum mixtures (100 μL/well) were then transferred onto confluent Vero cell monolayers cultured in 96-well plates and further incubated at 37°C under 5% CO_2_ for 7–10 days. Cells inoculated with virus-only (no serum) and cell-only (no virus) served as positive and negative controls, respectively. CPE was monitored daily under an inverted light microscope, and the neutralizing antibody titer was defined as the highest serum dilution that completely inhibited viral CPE in ≥50% of replicate wells. Statistical analysis was performed using GraphPad Prism v9.0, and geometric mean titers (GMT) with 95% confidence intervals were calculated.

### 2.10 Detection of IFN-γ and IL-2 secretion in vaccine-immunized mice

T-cell responses were determined by gamma interferon (IFN-γ) (MABTECH, 3321-4AST-2) and Interleukin-2 (IL-2) (MABTECH, 3441-4APW-2) enzyme-linked immunosorbent spot (ELISpot) assay according to the manufacturer’s protocol. Spleens were harvested from immunized mice after booster immunization. Splenocytes were filtered through a 100 μm pore size nylon cell strainer (BD) and digested with red blood cell lysis buffer (Beyotime, C3702) to obtain a single-cell suspension. Splenocytes were seeded at 1×10^5^ cells/well in RPMI-1640 supplemented with 10% FBS and 1× P/S, pre-incubated at 37°C under 5% CO_2_ for 2 h, then stimulated with MuVs (1×10^5^ CCID_50_/mL each of genotypes A and F) and incubated for an additional 24 h under the same conditions. After stimulation, the cells were incubated with biotin-conjugated antibodies and streptavidin-HRP. Spots were developed using 3-amino-9 ethylcarbazole (AEC) substrate. The numbers of IFN-γ- and IL-2-secreting cells were counted using the automated ELISpot reader as described above. Data are presented as the number of spot-forming units (SFUs) per 10^5^ splenocytes.

### 2.11 Mumps virus neurovirulence assessment

One-day-old Lewis rats were intracranially inoculated using a microsyringe (Thermo Fisher, T_7011481529) equipped with an ultra-fine dual-wall needle (internal diameter: 0.15 mm; outer diameter: 0.72 mm). The viral suspension (0.01 mL containing 10³ CCID_50_) was slowly injected perpendicularly through the skull (27). Tested viral strains included: S79 vaccine, QBB-Vero-P2, QBB-2BS-3.2-P20, QBB-2BS-3.2-P30, QBB-2BS-3.2-P40, QBB-2BS-9.3-P20, QBB-2BS-9.3-P30, QBB-2BS-9.3-P40. Rats were euthanized 30 days post-inoculation, and brains were removed and fixed in 4% fixative solution (Solarbio, P1110) for histological analysis. Two 3 to 4 mm-thick sagittal slices were selected at a standard distance from either side of the anatomical midline from a fixed brain, paraffin-embedded, sectioned, and stained with hematoxylin and eosin. The severity of hydrocephalus was determined as the percentage of the total brain cross-sectional area (excluding the cerebellum) occupied by the lateral ventricle on each of the two sections per rat using Image Pro Plus image analysis software (Media Cybernetics, Silver Spring, Md.). The mean percentage of hydrocephalus in each experimental group of rats was calculated and designated as the rat neurovirulence test (RNVT) score (28).

### 2.12 Statistical analysis

Graphing and analysis were performed using the GraphPad Prism 8 Software (San Diego, CA, USA). Statistical significance was analyzed by one-way ANOVA. Data represent mean ± SEM (n = 3). Statistical significance was represented by asterisks and was marked correspondingly in the Figures: (ns; not significant, * *p* < 0.05, ** *p* < 0.01, *** *p* < 0.001).

## 3 Results

### 3.1 Isolation and identification of F genotype mumps virus QBB strains

**Figure 1:**
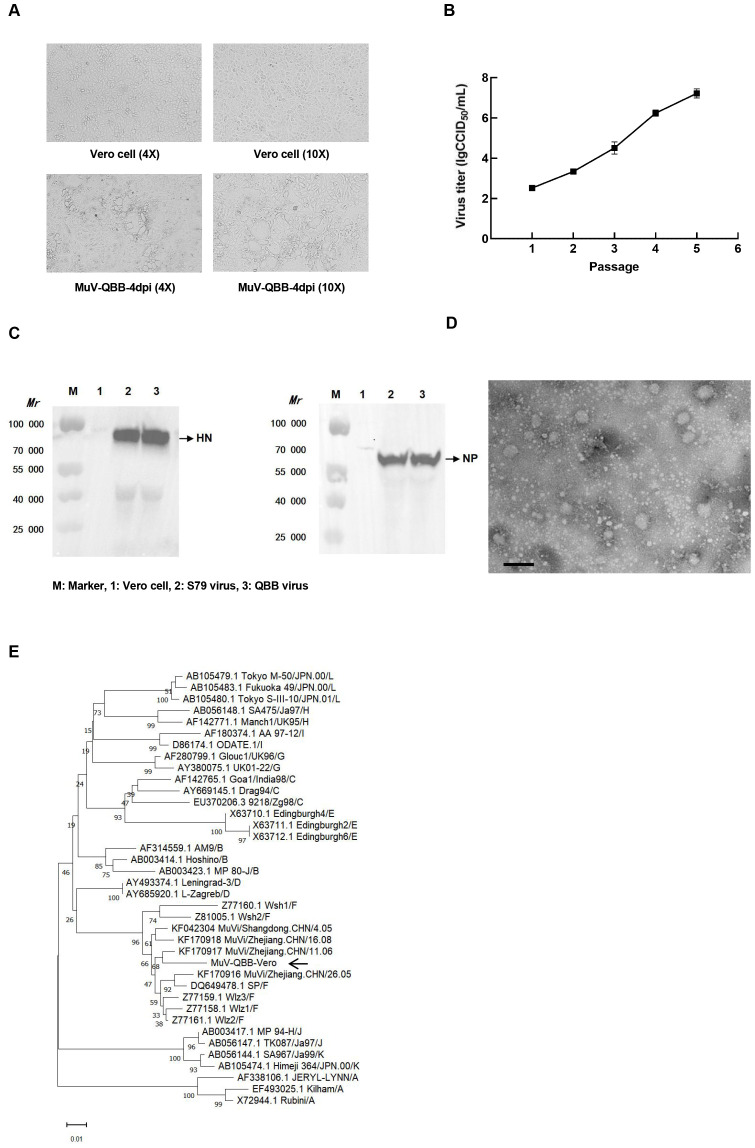
Isolation and identification of F genotype mumps virus QBB strains. **(A)** Cell morphology of Vero cells on day 4 post MuV-QBB infection. **(B)** Virus titer of different passages of MuV-QBB were detected in vero cells. **(C)** Western blot analysis of HN and NP proteins of the mumps. **(D)** Electron microscopy image of MuV- QBB. Scale bar: 500 nm. **(E)** Analysis and comparison the phylogenetic tree of SH and peripheral genes sequence of different genotypes mumps. Results are presented as means ± SD, n = 3.

Viral isolation from clinical samples of suspected mumps patients was performed in Vero cells, and one positive adaptive growth strain was obtained by screening from six samples. According to the principle of mumps strain nomenclature, the strain was named MuV/JiLin.CHN/2022 (QBB strain) (27). The whole genome sequence of the QBB strain is shown in **Supplementary Material S1**. As shown in **Figure 1A**, the morphology of Vero cells showed aggregation, rounded morphology, cellular aging, and shedding on day 4 post-infection. The viral titers increased progressively through serial passaging, reaching 7.20 lgCCID_50_/mL at passage 5 (P5) (**Figure 1B**). NP protein is the main structural protein of the MuV nucleocapsid, and HN protein is the main surface antigenic protein of MuV. As shown in **Figure 1C**, Western blot results showed that the amplicon bands of NP and HN proteins in MuV-QBB were consistent with S79 (positive control). Moreover, TEM revealed that the MuV-QBB virus exhibited a sphere-shaped morphology approximately 200 nm in diameter, which is in accordance with the structural characteristics of typical mumps virus (**Figure 1D**). The universal standard for MuV genotyping is based on the SH genesequences. To determine the genotypic classification of the MuV-QBB strain, we aligned the SH gene sequence with representative genotype F reference strains and constructed a neighbor-joining phylogenetic tree (2). As shown in **Figure 1E**, results of the evolutionary tree analysis showed that the MuV-QBB strain was located on the same branch as the Wshl, Wsh2, and Wlz1, Wlz2, Wlz3 and SP mumps strains (all F genotype mumps viruses), which indicated that MuV-QBB strains belonged to F genotype mumps viruses.

### 3.2 Preparation of MuVs 2BS cell adaptation strains

**Figure 2:**
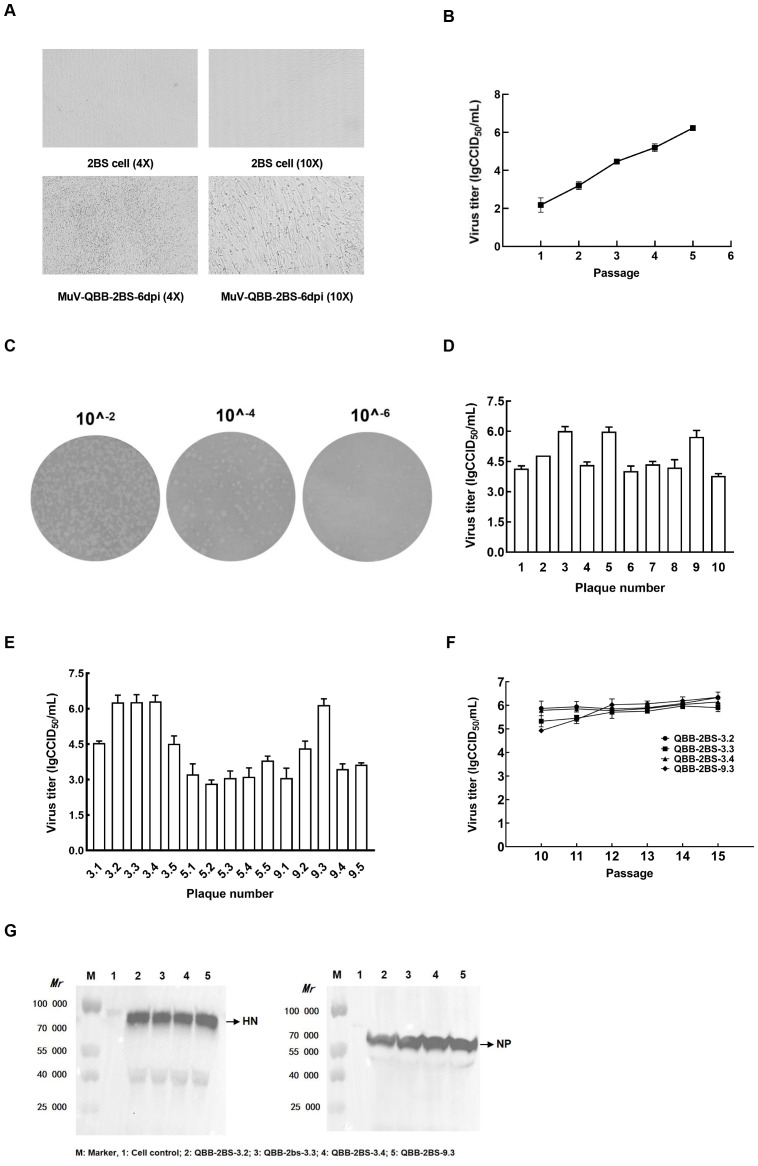
Generation of F genotype mumps virus 2BS cell-adapted strains. **(A)** Morphological changes in 2BS cells at 6 dpi with MuV-QBB. **(B)** Virus titer detection of MuV-QBB in 2BS cells at different passages. **(C)** Plaque clone of MuV-QBB mumps virus on 2BS cells. **(D)** Virus titer of the first round of plaque purification. **(E)** Virus titer of the second round of plaque purification. **(F)** Virus titer of four MuV-QBB 2BS cell-adapted strains from different passages. **(G)** Detection of HN and NP proteins by western blot in MuV-QBB 2BS-adapted strains. Results are presented as means ± SD, n = 3.

The MuV-QBB was passaged in 2BS cells up to P3, the cells exhibited typical cytopathic effects (CPE), whereas the negative control showed normal cell morphology (**Figure 2A**). As shown in **Figure 2B**, viral titer increased steadily, reaching 6.23 lgCCID_50_/mL at P5 generation. Subsequently, two rounds of plaque purification followed by viral amplification were performed. As shown in **Figure 2C**, plaques appeared round with smooth surfaces and well-defined edges at 7 days post-infection. The 3, 5, and 9 plaque numbers were selected from 10 plaques based on the viral titer after the first round of plaque purification (**Figure 2D**). In the second round, 5 plaques were screened respectively from the plaques obtained in the first round of screening (**Figure 2E**). The number of 3.2, 3.3, 3.4, and 9.3 plaques were taken based on the results of CPE and virus titers after passaging and inoculated on 2BS cells for culture. As shown in **Figure 2F**, the virus titers stabilized at 6.0 lgCCID_50_/mL after passaging to P15. Western Blot assay showed that HN and NP proteins were detected in all four MuV-QBB 2BS cell-adapted strains (**Figure 2G**).

### 3.3 Evaluation of immunogenicity of the QBB strain of MuVs in mice

**Figure 3:**
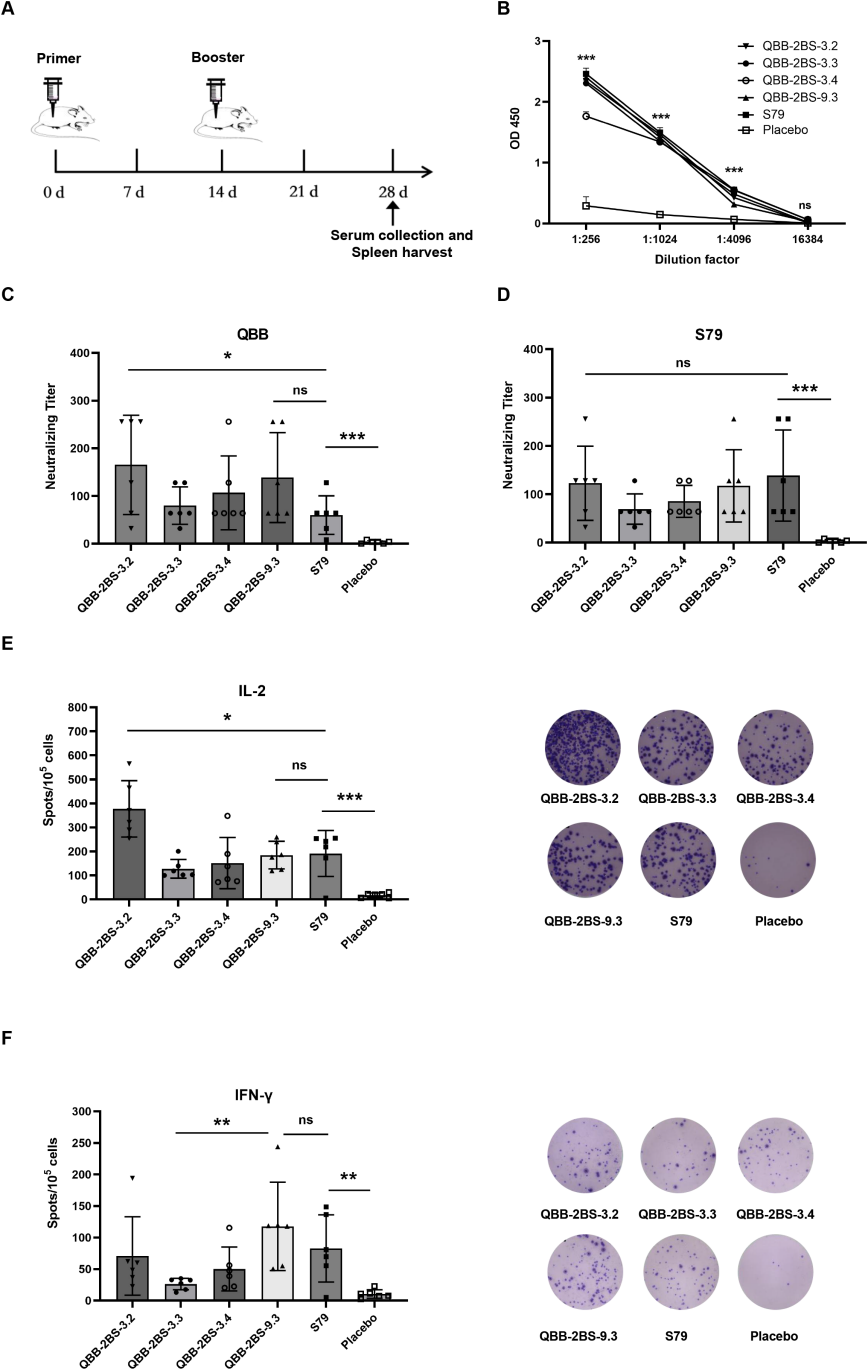
Evaluation of immunogenicity of the QBB strain of MuVs in mice. **(A)** Administration schedule of attenuated mumps vaccine candidates. **(B)** Total IgG elicited in BALB/c mice serum among different immune groups measured using ELISA. Results are presented as means ± SD, n = 3. ns; not significant, ***p < 0.001 (QBB-2BS-3.2 group compared to Placebo). **(C)** Serum neutralizing antibody titer against MuV-QBB. **(D)** Serum neutralizing antibody titer against S79. IL-2 **(E)** and IFN-γ **(F)** ELISpot analysis was performed and the spots were counted. ns; not significant, *p < 0.05, **p < 0.01, ***p < 0.001.

To evaluate the immunogenicity of the QBB candidate strains, BALB/c mice were immunized and tested for humoral and cellular immune responses. The immunization schedules are depicted in **Figure 3A**. As shown in **Figure 3B**, MuV-specific serum IgG levels, measured by ELISA, were significantly elevated in all immunized groups compared to the negative control group. Neutralization assay analysis of cross-protection conferred by the vaccine candidates revealed that mice vaccinated with QBB-2BS-3.2 developed significantly higher neutralizing antibody titers against the homologous QBB strain compared to those receiving the licensed S79 vaccine (**Figure 3C**), whereas neutralization titers against the heterologous S79 strain were comparable between both vaccine groups (**Figure 3D**). To explore the cellular immune response triggered by the QBB candidate strains, an ELISpot assay was performed to test IFN-γ and IL-2 using immunized BALB/c mice splenocytes stimulated with mixtures of MuVs. Immune responses indicate vaccine-induced responses detected in ELISpot IFN-γ and IL-2 assays. The levels of IL-2 were significantly higher in QBB-2BS-3.2 group compared to S79 group (**Figure 3E**), whereas the levels of IFN-γ were similar in QBB-2BS-9.3 and S79 group (**Figure 3F**).

### 3.4 Biological characteristics and genetic stability of the QBB strain of MuVs

**Figure 4:**
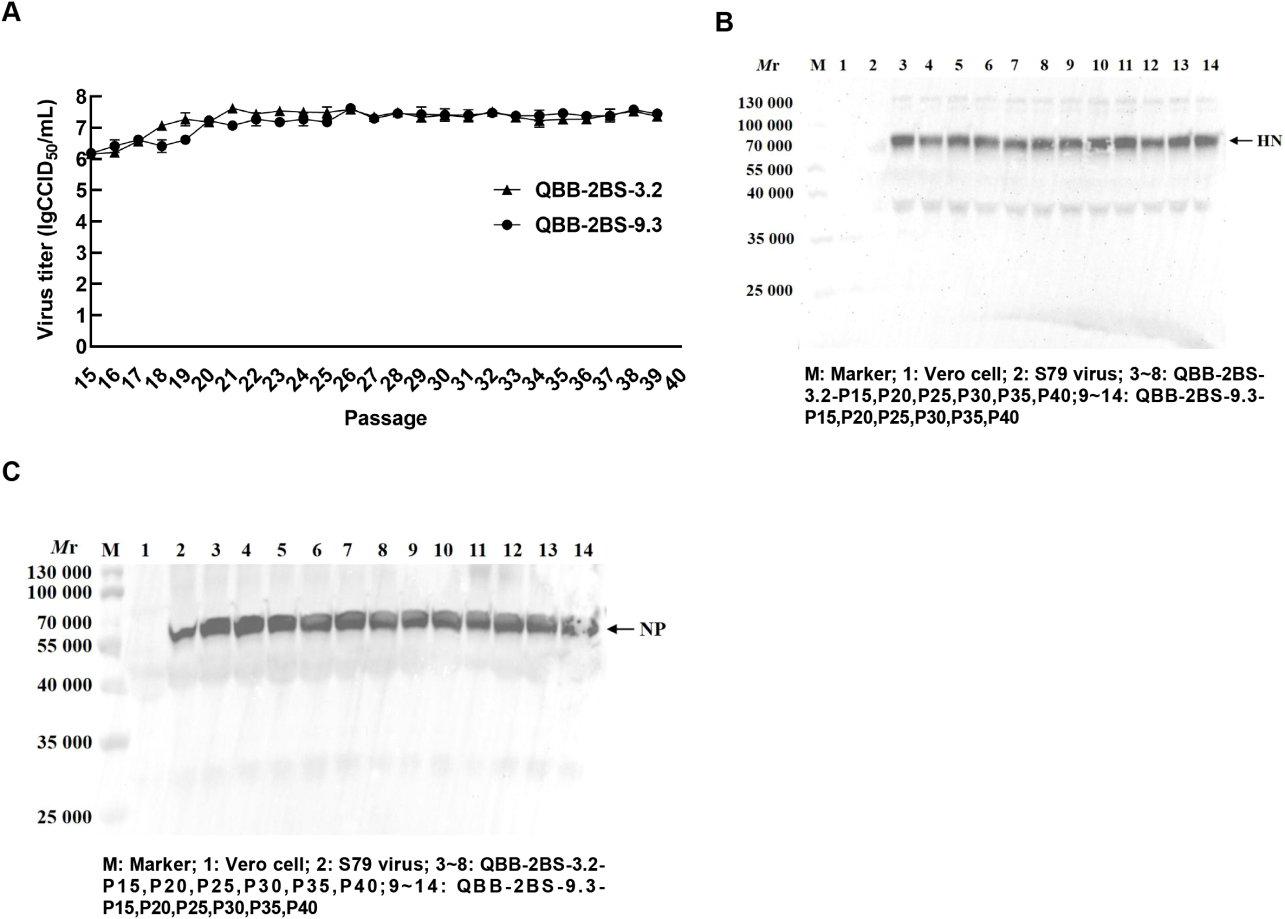
Biological characteristics and genetic stability of the QBB strain of MuVs. **(A)** The multiple growth curve of QBB-2BS-3.2 and QBB-2BS-9.3 strains from P15 to P40. Results are presented as means ± SD, n = 3. **(B)** Western blot of HN proteins in different passages of QBB strains. **(C)** Western blot of NP proteins in different passages of QBB strains.

To investigate the biological characteristics and genetic stability of the 2BS cell-adapted strain of F-genotype mumps virus (MuV). The QBB-2BS-3.2 and QBB-2BS-9.3 strains were serially passaged to P40 in 2BS cells, and virus titers were measured at P15, P20, P25, P30, P35, and P40. As shown in **Figure 4A**, the titers of the F genotype 2BS cell-adapted strains QBB-2BS-3.2 and QBB-2BS-9.3 gradually increased in the early generations and stabilized above 7.00 lgCCID_50_/mL after P20. Western blot analysis revealed stable expression of HN and NP proteins remained consistent across P15 to P40 for both QBB-2BS-3.2 and QBB-2BS-9.3 strains (**Figures 4B, C**). Whole-genome sequencing showed that the QBB-2BS-3.2 strain had 8 non-synonymous mutations (**Table 1**), and the QBB-2BS-9.3 strain had 7 non-synonymous mutations during P5 to P40 generations (**Table 2**).

**Table 1:** Comparison of whole genome sequences of different generations of QBB-2BS-3.2 strain.

Gene	Position	S79	QBB-2BS-3.2							Amino acids
			P5	P15	P20	P25	P30	P35	P40	
NP	74	T	T	C	C	C	C	C	C	I-T
M	200	G	G	A	A	A	A	A	A	V-I
F	194	A	A	G	G	G	G	G	G	T-A
	531	T	T	T	T	T	G	G	G	S-R
HN	347	T	T	C	C	C	C	C	C	T-H
	464	A	A	A	A	A	A	G	G	N-S
	526	A	G	A	A	A	A	A	A	K-E-K
L	1561	T	T	T	T	T	C	C	C	I-T

**Table 2:** Comparison of whole genome sequences of different generations of QBB-2BS-9.3 strain.

Gene	Position	S79	QBB-2BS-9.3							Amino acids
			P5	P15	P20	P25	P30	P35	P40	
M	171	G	G	G	G	A	A	A	A	C-Y
	200	G	G	G	G	A	A	A	A	V-I
F	194	A	A	G	G	G	G	G	G	T-A
HN	347	T	T	C	C	C	C	C	C	T-H
	466	A	G	G	G	G	A	A	A	N-S-N
	526	A	G	A	A	A	A	A	A	K-E-K
L	197	A	A	G	G	A	A	A	A	K-E

### 3.5 Evaluation of mumps virus neurovirulence in neonatal rat model

**Figure 5:**
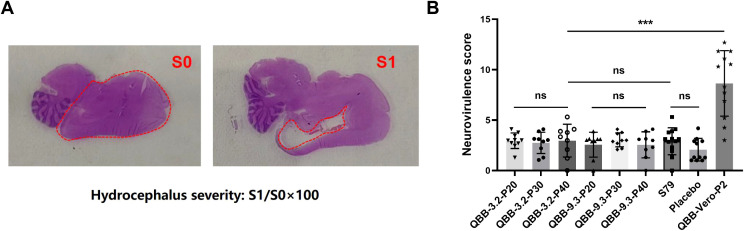
Evaluation of mumps virus neurovirulence in neonatal rat model. **(A)** HE staining of brain tissue of Lewis rats. **(B)** Neurovirulence score of rat hydrocephalus inoculated with MuVs candidates. ns; not significant, *p < 0.05, **p < 0.01, ***p < 0.001.

The neurovirulence of the candidate strains QBB-2BS-3.2 and QBB-2BS-9.3 was evaluated using the neonatal rat model. As shown in **Figure 5**, the left panel shows HE staining images of brain tissue from mice without hydrocephalus, and the right panel shows HE staining images of brain tissue from mice with hydrocephalus. The mean S/S0 ratio is the hydrocephalus neurotoxicity score. As shown in **Figure 5B**, there was no significant difference in cerebral neurotoxicity scores of QBB-2BS-3.2 and QBB-2BS-9.3 strains in all generations compared with the S79 vaccine group or the placebo group, and on the contrary, when compared with the QBB-Vero-P2 group. This indicates that the neurotoxicity of QBB-2BS-3.2 and QBB-2BS-9.3 was significantly weakened after adaptation, and there was no virulence reversion with the increase in the number of generations.

## 4 Discussion

Vaccines are an important means of preventing and controlling the spread of disease. Live attenuated vaccines are of great interest because of their long-lasting immunity and long duration of action, e.g., attenuated polio vaccine, attenuated chickenpox vaccine, and measles-mumps-rubella (MMR) vaccine, which have shown remarkable results in combating viral infections. The attenuated mumps vaccine was incorporated into China in the 1990s, and formally integrated into the National Expanded Programme on Immunization (EPI) in 2008. The most widely used vaccine strains in China are S79 and WM84, based on the JL strain (A genotype). There is only one serotype of the mumps virus, and virus strains of different genotypes have cross-protective effects. However, in the last decade, we have encountered repeated outbreaks of mumps in highly vaccinated populations, which call into question the effectiveness of available vaccines (29–33). Thus, vaccine strains and vaccination strategies may need to be further evaluated and optimized to ensure broader protection (34–36). Moreover, exploring the genetic characterization of mumps viruses and gaining insight into the immunogenicity differences between genotypes are essential to guide the design and use of mumps virus vaccines (37, 38).

In this study, six specimens of throat swabs were collected from clinical samples of mumps patients after decontamination and filtration and inoculated with Vero cells, of which one sample (MuV-QBB) was screened. Western blot bands showing NP and HN protein production in MuV-QBB and control S79 group (**Figure 1C**). Meanwhile, TEM results showed that MuV-QBB virus was a globular structure with a diameter of about 200 nm, a typical mumps virus morphological structure (**Figure 1D**). The SH gene sequences are usually used as mumps virus genotyping criteria. We analyzed the homology of the SH gene sequences of the MuV-QBB strain with the corresponding sequences of the representative strains of known genotypes, and constructed a gene kinship tree by using the neighbor-joining method, and the results of the evolutionary tree analysis showed that the MuV-QBB strain was located on the same branch as Wshl, Wsh2, Wlz1, Wlz2, Wlz3, and SP. Wshl, Wlz1, Wlz2, Wlz3, SP, and other F genotype mumps strains were located in the same branch with high homology (**Figure 1E**), indicating that the MuV-QBB strain belongs to the F genotype mumps virus.

Cell lines used for vaccine production are increasingly becoming the focus of researchers. The WHO recommends Human diploid cell strains (HDCSs) as the safest cell culture substrate for the production of viral vaccines, and they have become the preferred cell substrate for vaccine production worldwide (39, 40). Mumps live attenuated vaccine is mainly produced using chicken embryo cells as a heterologous cellular lines, which may cause allergic reactions, and chicken embryo cells carrying infectious avian retroviruses may cause side effects. The human embryonic lung diploid cell 2BS strain is derived from healthy human embryonic lung tissue, which has the potential for large-scale culture and has a good safety profile (41). Currently, 2BS cells have been successfully produced in hepatitis A, polio, rubella, and varicella vaccines (42, 43). MuV-QBB virus was inoculated into 2BS cells, and after two rounds of plaque purification, four MuV-QBB adaptor strains were successfully screened. The viral titer was stabilized above 6.0 lgCCID_50_/mL when the strains were passaged to P15 (**Figure 2F**). Western blot bands showing NP and HN protein production in four MuV-QBB-adapted strains (**Figure 2G**).

To evaluate the immunogenicity of the QBB candidate strains, BALB/c mice were immunized and tested for humoral and cellular immune responses. ELISA using the MuV-specific serum antibodies revealed that the total IgG levels was higher in all immunized groups than in the negative control group, but there was no significant difference among the six experimental groups (**Figure 3B**). Studies have shown that cross-protection between genotypes is limited. Consistent with reports of genotype-restricted cross-protection, our neutralization assays demonstrated that while QBB-2BS-3.2 induced significantly higher F-genotype-specific neutralizing titers than S79 (**Figure 3C**), both candidates maintained comparable neutralization against the homologous S79 strain (**Figure 3D**), mirroring the limited heterotypic immunity observed in Jeryl Lynn derived vaccines. To explore the cellular immune response triggered by the QBB candidate strains, an ELISpot assay was performed to test IFN-γ and IL-2 using immunized BALB/c mice splenocytes stimulated with mixtures of MuVs (44). IFN-γ serves as the central mediator of protective immunity against mumps virus by directly activating the viral clearance capacity of macrophages, enhancing antigen presentation, and inducing antiviral protein expression; whereas IL-2 primarily marks Th1 cell activation and indirectly supports immune responses by promoting T cell expansion (45). The levels of IFN-γ and IL-2 were significantly elevated in experimental groups compared to the placebo group. Moreover, the levels of IL-2 were significantly higher in QBB-2BS-3.2 groups compared to S79 group (**Figure 3E**), and the levels of IFN-γ were similar in QBB-2BS-9.3 and S79 groups (**Figure 3F**). Therefore, QBB-2BS-3.2 and QBB-2BS-9.3 were selected for subsequent genetic stability studies.

To investigate the biological characteristics and genetic stability of the 2BS cell-adapted strain of F-genotype mumps virus (MuV). The QBB-2BS-3.2 and QBB-2BS-9.3 strains were adapted to 40 passages in 2BS cells. The titers of the F-genotype 2BS cell-adapted strains QBB-2BS-3.2 and QBB-2BS-9.3 gradually increased from passage 15 to 40, and stabilized at 7.40 lgCCID_50_/mL (**Figure 4A**). The expression levels of HN and NP proteins remained consistent across passages 15 to 40 for both QBB-2BS-3.2 and QBB-2BS-9.3 strains (**Figures 4B, C**). Whole-genome sequences showed that eight non-synonymous mutations occurred in QBB-2BS-3.2 and seven non-synonymous mutations occurred in QBB-2BS-3.2 during the passaging, compared with strain QBB-2BS-P5 (**Tables 1**, **2**). It has been reported that the aa464 site of the HN protein was associated with viral neurotoxicity; Cui et al. showed that amino acids located at sites 329-340, 354, and 356 on the HN protein were associated with cross-neutralizing ability (46). Malik et al. confirmed that amino acid changes in the aa466 of the HN protein were associated with alterations in neurotoxicity (47, 48). Previous studies have confirmed that the aa464 (N-K) mutation in the HN protein of the Urabe AM9 vaccine strain enhances neurovirulence, whereas the aa466 (S-N) mutation in the 88–1961 strain reduces neurovirulence (46). Based on these findings, we will employ reverse genetics techniques to generate infectious clones harboring these specific mutations and conduct neuroinvasive phenotype analysis in neuronal models. The correlation of amino acid mutations occurring during the transmission of the two candidate strains with the antigenicity and neurotoxicity of MuV remains to be investigated.

Neurotoxicity is one of the important indexes used to evaluate the safety of live attenuated MuV vaccines. Rhesus monkeys are commonly evaluated for MuV live attenuated vaccine neurotoxicity. However, it has been shown that mumps vaccine candidates assessed as safe by monkey neurotoxicity modeling can still cause meningitis and encephalitis in clinical trials (40). Rubin et al. proposed that neuropathology in neonatal rats inoculated with mumps virus could serve as a sensitive indicator of the neurovirulence potential of the human central nervous system, which could differentiate between wild-type (WT) virus strains and vaccine strains (23, 28, 49, 50). Subsequently, an international collaborative study conducted by the U.S. Food and Drug Administration (FDA) and the National Institute for Biological Standards and Control (NIBSC) evaluated the reliability, robustness, and reproducibility of the rat-based neurovirulence test (RNVT) in assessing the neurovirulence potential of MuV in humans (51). In this study, the neurotoxicity of QBB-2BS-3.2 and QBB-2BS-9.3 was evaluated using a neonatal Lewis suckling rat model. Results showed that no significant difference in RNVT score of QBB-2BS-3.2 and QBB-2BS-9.3 adapted strains in all generations compared with the vaccine group S79 or the placebo group (**Figure 5B**), indicating that the neurotoxicity of QBB-2BS-3.2 and QBB-2BS-9.3 was attenuated and can be used as candidate strains. Notably, the neurovirulence stability observed between p20 and p40, in contrast to the significant attenuation seen between p2 and p20, suggests that genetic adaptations leading to reduced neurovirulence are selected during early serial passaging, eventually reaching a stable plateau. In follow-up studies, we will analyze the whole genome sequencing results of key passaged virus populations to identify mutation sites associated with neurotoxicity attenuation, followed by functional validation using reverse genetics to characterize their specific effects on neurovirulence.

In conclusion, the F genotype mumps QBB strains were successfully isolated from clinical samples, and two MuV 2BS cell-adapted strains were screened by strain inoculation with 2BS cell-adapted cultures and plaque purification. In addition, the candidate strains were tested for genetic stability, immunogenicity, and neurotoxicity. These results provide a basis for the development of a live attenuated F genotype MuV vaccine.

## Data Availability

The datasets presented in this study can be found in online repositories. The names of the repository/repositories and accession number(s) can be found in the article/**Supplementary Material**.
