# Supplementary material for: Development and characterization of the genotype F attenuated mumps candidate strains
Source: Front Immunol. 2025 Jul 22;16:1629585. doi: 10.3389/fimmu.2025.1629585 (PMC12321783; doi:10.3389/fimmu.2025.1629585)
Supplement: Supplementary file 1 [file Table1.docx]

**Supplementary data**

**S1. The whole genome sequence of MuV/JiLin.CHN/2022 (QBB strain)**

ACCAAGGGGAAAATGAAGATGGGATATCGGTAGAACAAATAGTGTAAGAAACAGTAAGCCCGGAAGTGGTGTTTTGCGATTTCGAGGCCGGGCTCGATCCTCACCTTCCATTGTCGATAGAGGATATTTTGACACTATCTGAAAAATGTCGTCTGTGCTCAAAGCATTTGAGCGATTCACGATAGAACAGGAACTCCAGGACAGGGGTGAGGAGGGTTCAATTCCGCCGGAGACTTTAAAGTCAGCAGTCAAAGTCTTCGTCATTAACACACCCAATCCCACCACACGCTACCAGATGCTAAACTTTTGCCTAAGAATAATCTGCAGTCAAAATGCTAGGGCATCTCACAGGGTAGGTGCATTGATAACATTATTCTCACTTCCCTCAGCAGGCATGCAAAATCATATTAGACTAGCAGATAGATCACCCGAGGCTCAGATAGAACGCTGTGAGATTGATGGTTTTGAGCCCGGCACATATAGGTTGATTCCGAATGCACGCGCCAATCTTACTGCCAATGAAATTGCCGCCTATGCTTTGCTTGCAGATGACCTCCCTCCAACCATAAATAATGGAACTCCTTATGTACATGCAGATGTTGAAGGTCAGCCATGTGATGAGATTGAGCAATTCCTGGATCGATGCTACAGTGTACTGATCCAGGCTTGGGTGATGGTCTGTAAATGTATGACAGCGTACGACCAACCTGCTGGATCTGCTGATCGACGATTTGCTAAGTACCAGCAGCAAGGTCGCCTAGAAGCAAGATACATGCTGCAGCCGGAGGCCCAAAGGTTGATTCAAACTGCCATCAGGAAAAGTCTTGTTGTTAGACAGTACCTTACCTTCGAACTCCAATTGGCAAGACGGCAGGGATTGCTATCAAACAGATACTATGCAATGGTAGGTGACATTGGAAAGTACATTGAGAATTCAGGCCTTACTGCCTTCTTTCTCACCCTCAAATACGCACTAGGTACTAAATGGAGTCCTTTGTCATTGGCCGCATTCACAGGTGAACTCACTAAGCTCCGATCCTTGATGATGTTGTATCGGGATCTCGGGGAACAAGCCAGATACCTTGCTTTGTTGGAGGCTCCCCAAATAATGGACTTTGCACCTGGGGGCTACCCATTGATATTCAGTTATGCCATGGGAGTCGGTACAGTCCTAGATGTCCAGATGCGAAATTACACTTATGCACGACCTTTCCTAAATGGCTATTACTTCCAGATTGGGGTTGAGACCGCAAGACGGCAACAAGGCACTGTTGACAACAGAGTAGCAGATGATCTAGGCCTGACTCCTGAGCAAAGAACTGAGGTTACTCAGCTTGTTGACAGGCTTGCAAGGGGCAGAGGTGCAGGGATACCAGGTGGGCCTGTGAATCCATTTGTTCCTCCAGTTCAACAGCAACAACCTGCTGCTGTATATGAGGACATTCCTGCATTGGAGGAATCAGATGACGATGGCGATGAAGATGGAGATGCAGGATTCCAAAATGGAGCACAAGCACCAGCTGTAAGACAGGGAGGTCAAAATGACTTCAGAGTACAGCCACTACAGGATCCAATTCAAGCACAGCTTTTCATGCCACTATATCCTCAAGTCAGCAACATCCCAAATCATCAGAATCATCAGATCAATCGCATCGGGGGGATGGAACACCAAGATTTATTACGATATAACGAGAATGGTGATTCTCAACAAGACGCGAGGGGCGAACAAGGAAATACCTTCCCAAACAATCCTAATCAAAACACACAGTCGCAAGTGGGCGACTGGGATGAGTAAATTACTGACATGGTCAGACTACCCCCAAATGCAATTACCCCAGGACAATCTAACCACAGCTAACTGCCCAGATCCACTACATTCCATCAATATTTAGCCTTTAAGAAAAAATTAGGCCCGGAAAGAATTAGTTCTACGAACATCGACAAAGTTATCTTGATCGTGTTTCTTTCCGGGCAAGCCATGGATCAATTTATAAAACAAGATGAAACTGGTGATTTAATTGAGACAGGAATGAACGTTGCAAATCATTTCCTATCCGCCCCCATTCAGGGAACCAACTCGTTGGGCAAAGCCACAATCATCCCTGGTGTTGCACCAGTACTCATTGGCAATCCAGATCAAAAGAACATTCAATACCCCACCGCATCACATCAGGGATCCAAGTCAAAGGGCAGAAGCTCAGGGACCAGGCCCATCATAGTCTCATCTTCCGAAGGGGGCACTGGAGGGACTCAGATTCCTGAGCCCCTCTTCGCACAAACAGGGCAGGGTGGCATTGTCACCACCGTTTATCAGGATCCAACTATCCAACCAACAGGTTCATACCGAAGTGTAGAATTGGCTAAGATAGGAAAAGAGAGAATGATTAATCGATTTGTTGAAAAACCAAGGACCTCAACGCCGGTGACAGAATTTAAGAGGGGGGCCGGGAGCGGCTGCTCAAGGCCAGATAATCCAAGAGGAGGACATAGACGGGAATGGAGCCTCAGCTGGGTCCAAGGAGAGGTCCGGGTCTTTGAGTGGTGCAACCCCATATGCTCACCTATCACTGCCGCAGCAAGATTCCACTCCTGCAAATGTGGGAATTGCCCCGCAAAGTGCGATCAGTGCGAACGAGATTATGGACCTCCTTAGAGGGATGGATGCTCGTCTGCAACATCTTGAACAAAAGGTGGACAAGGTGCTTGCACAGGGCAGCATGGTGACCCAAATAAAGAATGAATTATCAACAGTAAAGACAACACTAGCTACAATTGAGGGAATGATGGCGACCGTAAAGATCATGGATCCTGGAAACCCGACAGGGGTCCCAGTCGATGAGCTTAGAAGAAGTTTCAGTGATCATGTAACAATTATTAGTGGACCAGGAGATGTGTCATTCAGCTCAGGTGAAGAGCCTACACTGTATTTAGATGAACTAGCAAGGCCTGTCCCCAAGCCTCGTCCTGCAAAGCAGCCAAAACCCCAACCAGTAAAGGATTTAGCAGGACGGAAAGTGATGATAACCAAAATGATCACTGACTGTGTGGCCAACCCTCAAATGAAGCAGGTGTTTGAGCAACGACTGGCAAAGGCCAGCACGGAGGATGCCCTGAATGACATCAAGCGAGACATCATACGGAGCGCCATATGAATCAACCAGAAACACAAGACTTACAGGAAAATCCACAGCCTAAGAGCCACAATGATTCCCTGTTAAATAAAAAATAAGCACGAACACAAGTCCGATCCAACCATAGCAGCAATGGCCGGATCACAGATCAAAATTCCTCTTCCAAAGCCCCCCGATTCAGACTCCCAAAGACTAAATGCATTCCCTGTAATTATGGCCCAAGAAGGTAAAGGGCGACTCCTCAGACAGATCAGACTTAGGAAAATATTATCAGGGGATCCGTCTGATCAGCAAATTACATTTGTGAATACATATGGATTCATCCGTGCCACTCCAGAAACATCCGAGTTCATCTCTGAATCATCACAACAAAAGGTGACTCCTGTAGTGACGGCGTGCATGCTGTCCTTCGGTGCTGGACCAGTACTAGAAGACCCACAACATATGCTGAAAGCTCTTGACCAGACAGACATCAGGGTTCGGAAAACAGCGAGTGATAAAGAGCAGATCTTATTCGAGATCAACCGTATCCCCAATCTATTCAGGCATCATCAAATATCTGCGGACCATCTGATTCAGGCCAGCTCCGATAAATATGTCAAGTCACCAGCAAAGTTGATTGCAGGAGTAAATTACATTTACTGTGTCACATTTTTATCTGTGACAGTTTGCTCTGCCTCACTCAAGTTTCGAGTTGCGCGCCCATTGCTTGCTGCACGATCTAGATTAGTAAGAGCAGTTCAGATGGAAGTTTTGCTTCGGGTAACTTGCAAGAAAGATTCCCAAATGGCAAAGAGCATGTTAAATGACCCTGATGGCGAAGGGTGCATTGCATCCGTGTGGTTCCACCTGTGTAATCTGTGCAAAGGCAGGAATAAACTTAGAAGTTATGATGAAAATTATTTTGCATCTAAATGCCGTAAGATGAATCTGACAGTCAGCATAGGAGACATGTGGGGGCCAACCATTCTAGTCCATGCAGGCGGTCACATTCCGACAACTGCAAAACCTTTCTTCAACTCAAGAGGCTGGGTCAGCCACCCCATCCACCAATCATCACCATCGTTGGCGAAGACCCTATGGTCATCTGGGTGCGAAATCAAGGCTGCCAGTGCTATCCTCCAGGGCTCAGATTATGCATCACTCGCAAAAACTGATGACATAATATACTCAAAGATAAAAGTCGATAAAGATGCTGCCAACTACAAGGGTGTATCCTGGAGTCCATTCAGGAAGTCTGCCTCAATGAGCAACCTATGAAAATTTCCTCTATTCCCACTGATGCCTCCAGGAGGATCAACAATCAGTCTGATTTGACTGGTGGTAACTTGATTGAAATTATAGAAAAAATAAGCCTAGAAGGATATCTTACTTCTCGACTTTCCAACTTTGAAAAGAGAATTAATCAGTAATCATGAAGGCTTTTTTAGTTATTTGCTTGGGCTTTGCAGTCTTTTCATATTCTATTTGTGTGAATATCAACATCTTGCAGCAAGTTGGATATATCAAGCAACAAGTCAGGCAACTAAGCTATTACTCACAAAGTTCAAGCTCCTACATAGTGGTCAAGCTTTTACCGAATATCCAACCCACTGATGACAGCTGTGAATTTAAGAGTGTAACTCAATACAATAAGACCTTGAGTAATTTGCTCCTTCCAATTGCAGAAAACATAAACAATATTGCATCGCCCTCACCTGGGTCAAGACGTCATAAAAGGTTTGCTGGCATTGCCATCGGCATTGCTGCGCTCGGTGTTGCGACTGCAGCACAAGTAACTGCCGCTGTCTCATTAGTTCAAGCACAGACAAATGCACGTGCGATAGCGGCGATGAAAAATTCAATACAGGCAACTAATCGAGCAGTCTTCGAAGTGAAAGAAGGAACTCAACAGTTAGCTATAGCGGTACAAGCAATACAAGACCACATCAATACTATTATGAACACTCAATTGAGCAATATGTCTTGTCAGATCCTTGATAACCAGCTTGCGACTTCCCTAGGATTATACCTAACAGAATTAACAACAGTGTTTCAGCCCCAATTAATTAATCCGGCACTGTCACCGATTAGTATACAAGCCTTGAGGTCTTTGCTTGGAAGTATGACACCTGCAGTGGTTCAAGCAACATTATCTACTTCAATTTCTGCTGCGGAAATACTAAGTGCCGGTCTAATGGAGGGTCAGATTGTGTCTGTTCTGCTAGATGAGATGCAGATGATAGTTAAGATAAATATTCCAACCATTGTCACACAATCAAATGCATTGGTGATTGACTTCTACTCAATTTCGAGCTTTATTAATAATCAGGAAACCATAATTCAATTACCAGACAGGGTCTTGGAGATCGGGAATGAACAGTGGAGCTATCCAGCTAAAAATTGTAAGTTGACAAGACACAACATATTCTGCCAATACAATGAGGCAGAGAGGCTGAGCCTAGAGTCAAAACTATGCCTTGCAGGAAATATAAGTGCCTGTGTGTTCTCACCCATAGCAGGAAGTTATATGAGGCGATTTGTAGCACTGGATGGAACAATTGTTGCAAACTGTCGAAGTCTAACGTGTCTATGCAAGAGTCCATCTTATCCTATATACCAACCTGACCATCATGCAGTCACAACCGTTGATCTAACCGCATGTCAAACATTGTCCCTAGACGGATTAGATTTCAGTATTGTCTCTCTAAGCAACATCACTTATGCTGAGAACCTTACCATTTCATTGTCTCAGACAATCAATACTCAACCCATTGACATATCAACTGAACTAAGTAAAGTTAATGCATCCCTCCAAAATGCCGTTAAGTACATAAAAGAGAGCAACCAACAACTCCAATCTGTGAGTGTAAATTCCAAAATCGGAGCTATAATTCTAGCAGCCTTAGTTTTGAGCATCCTGTCAATCATCATTTCGCTATTGTTTTGCTGCTGGGCTTACATTGCAACTAAAGAAATCAGGAGAATCAACTTCAAAACAAATCATATCAACACAATATCAAGTAGTGTCGATGATCTCATCAGGTACTAATCCTAACATTGTGATTCATTCTGCATTGAGAAAAGATTTAGAAAAAAACCAAATTAAGAATGAATCTCCCGGGGTCGTAACGTCTCGTGACCCTGCCGTTGCACTATGCCGGCAATCCACCCTCCCTTATACCTAGCATTTCTATTGCTAATTCTTCTTCATCTGATCATAAATTTGTATGTCCGGATTATGCTAACCATTACTCACAAGACTGCGGTGCAACATGCAGCACTGTACCAGAGATCCCTCTTTCGTTGGAGTTTCGATCACTCACTCTAGAAAGATCTCCAACCCGGACAAGTCCCAATCCATCATGAGAGAACAGGCTGCATTCAAATAATGCTGTTCAATCATGAGACATAAAGAAAAAAGCAAGCCAGAACAAACTTAGGATCACAACACAACACAAAATATTAGCTGCTATCACAACTGTGCTCCAGCAGCTTGAAAGATGGAGCCCTCGAAATTCTTCACCATATCGGACAATGCCACCTTTGCACCTGGGCCTGTTATCAATGCAGCTGACAAGAAGACATTCCGAACCTGCTTCCGAATATTGGTACTGTCTGTACAAGCTGTTACCCTTATATTAGTCATTGTCACTTTAGGTGAGCTTGTGAGGATGATCAATGATCAAGGCTTGAGTAATCAGTTGTCTTCAATTACAAACAAGATAAGAGAGTCAGCTACTATGATTGCATCTGCTGTGGGAGTAATGAATCAAGTTATTCACGGTGTAACGATATCCTTACCCCTACAACTTGAGGGAAACCAAAATCAATTGTTATCCACACTTGCCACAATCTGTACAAGCAAAAAGCAAGTCTCAAACTGCTCTACGAACATCCCCTTAGTTAATGATCTTAGGTTTATAAATGGGATCAATAAATTCATCATTGAAGATTATGCAACTCATGATTTCTCTATCGGCCATCCACTCAACATGCCTAGCTTTATCCCAACTGCAACCTCACCCAATGGTTGCACAAGGATTCCATCCTTCTCTCTAGGTAAGACACACTGGTGCTACACACATAATGTAATTAATGCCAACTGTAAGGATCATACTTCGTCTAACCAATATGTTTCTATGGGGATTCTCGTTCAGACCGCGTCAGGGTATCCTATGTTCAAAACCTTAAAAATCCAATATCTCAGTGATGGCCTGAATCGGAAAAGCTGCTCAATTGCAACAGTCCCTGATGGATGCGCAATGTATTGTTACGTCTCAACTCAGCTTGAAACCGACGACTATGCAGGGTCCAGCCCACCTACCCAGAAACTTACCCTGTTATTCTATAATGACACCGTCACAGAAAGGACAATATCTCCATATGGTCTTGAAGGGAATTGGGCTACTTTGGTACCAGGAGTGGGGAGTGGAATATATTTCGAGAATAAATTGATCTTTCCTGCGTATGGGGGTGTCTTGCCCAATAGTACACTTGGAGTTAAATCAGCAAGAGAATTTTTCCGACCTGTTAACCCATATAATCCATGTTCAGGACCACAACAAGATTTAGATCAGCGTGCTTTGGGATCATACTTCCCAAGTTACTTCTCTAATCGAAGAGTGCAGAGTGCATTTCTTGTCTGTGCCTGGAATCAGATTCTAGTTACAAATTGCGAGCTAGTTGTCCCCTCAAACAATCAGACACTGATGGGTGCAGAAGGAAGAGTTTTATTGATCAATAATCGATTATTATATTATCAGAGAAGTACCAGCTGGTGGCCGTATGAACTCCTCTATGAGATATCATTCACCTTTACAAACTCTGGTCAATCATCTGTGAACATGTCCTGGATACCTATATACTCATTCACTCGTCCTGGTTCAGGTAACTGCAGTGGTGAAAATGTGTGCCCGACTGCTTGTGTGTCAGGGGTTTATCTTGACCCTTGGCCATTAACTCCATATAGCCACCAATCAGGCATTAACAGAAATTTCTATTTCACAGGTGCACTATTAAATTCAAGCACAACTAGAGTAAATCCTACCCTTTATGTCTCTGCCCTTAATAATCTTAAAGTACTAGCCCCATATGGTACTCAAGGACTGTTTGCCTCGTACACCACAACCACCTGCTTTCAAGATACCGGTGATGCTAGTGTGTATTGTGTTTATATTATGGAACTAGCATCGAATATCGTTGGAGAATTCCAAATTCTACCTGTGCTAACCAGACTGACCATCACTTGAGTCATAGTGAATGCAGCGGTAGGCCCTATGGGCGTGTCTCAAGTTTTATCGATTATTAAGAAAAAACAGGCCAGAATGGCGGGCCTAAATGAGATACTCTTACCTGAAGTACATTTGAACTCACCCATCGTTAGATATAAGCTTTTCTACTATATATTGCATGGCCAGTTACCAAATGATTTGGAGCCAGATGACTTAGGCCCACTAGCAAATCAGAATTGGAAGGCAATTCGATCTGAAGAATCCCAGGTTCATGCACGTTTAAAACAGATCAGAGTAGAACTCATTGCAAGGATTCCTAGTCTCCGGTGGACCCGCTCTCAAAGAGAGATTGCCATACTCATTTGGCCAAGAATACTTCCAATCCTGCAAGCATATGATCTTCGGCAAAGTATGCAATTGCCTACAGCATGGGAGAAATTGACTCAATCCACAGTTAATCTTATAAGTGATGGCCTAGCACGGGTTGTATTACATATAAGCAATCAACTGACAGGCAAGCCTAACTTGTTTACCAGATCCCGAGCAGGACAAGACGCAAAGGATTCCTCAATTCCATCCACTAGAGAGCTATCTCAAATATGGTTTAACAATGAGTGGAGTGGATCTGTAAAGACCTGGCTTATGATTAAATATAGAATGAGGCAGCTAATCACAAACCAAAAGACAGGTGAATTAACAGATTTAGTAACCATTGTGGATACTAGATCCACTCTATGCATTATTACCCCAGAATTAGTTGCTTTATACTCCAATGAGCACAAAGCATTAACGTACCTCACCTTTGAAATGGTCTTAATGGTCACTGATATGTTAGAAGGACGACTGAATGTTTCTTCTTTATGCACTGCTAGTCATTATCTGTCCCCTCTAAAGAAGAGAATTGAAATTCTTCTAACATTAGTTGATGACCTTGCTCTACTCATGGGGGATAAAGTATACGGTGTTGTCTCTTCACTTGAGAGTTTTGTTTATGCCCAATTACAATATGGTGATCCTGTTGTAGACATTAAGGGCACATTTTATGGATTTATATGTAATGAAATTCTCGACCTATTAACTGAAGACAACATCTTTACTGAAGAGGAGGCAAACAAGGTTCTTCTGGACTTGACATCACAGTTTGACAATCTATCCCCTGATTTAACTGCTGAGCTCCTCTGCATTATGAGACTTTGGGGCCATCCCACATTAACCGCCAGCCAAGCAGCATCCAAGGTCCGAGAGTCCATGTGCGCTCCCAAGGTATTAGATTTCCAAACAATAATGAAGACCCTGGCTTTCTTTCACGCAATCCTGATTAACGGTTATAGGAGGAGCCATAATGGAATCTGGCCTCCTACTACTCTTCATGGCAATGCCCCCAAAAGCCTCATTGAGATGCGTCATGATAACTCAGAGCTTAAGTATGAATATGTCCTCAAGAATTGGAAAAGTATATCTATGTTAAGGATACATAAATGCTTTGATGCATCACCTGATGAAGATCTCAGCATATTCATGAAGGATAAGGCAATAAGCTGTCCAAAGCAAGACTGGATGGGAGTATTTAGGAGGAGCCTGATAAAACAGCGATATCGTGATGCGAATCGACCTCTACCACAACCATTCAACCGACGGCTACTGTTGAATTTTCTAGAGGATGACAGATTCGATCCTATTAAAGAACTTGAGTATGTCACCAGTGGAGAGTATCTTAGGGACCCTGAATTTTGTGCATCTTACTCTCTCAAGGAGAAGGAGATAAAGGCTACAGGTCGTATATTTGCTAAAATGACAAAGAGAATGAGATCGTGCCAAGTAATTGCAGAGTCATTGTTGGCCAATCATGCAGGAAAATTAATGAGAGAGAATGGAGTTGTCTTAGACCAGTTAAAACTGACAAAATCTTTGTTAACGATGAACCAAATTGGTATTATATCGGAGCACAGCCGAAGATCCACTGCTGACAACATGACTTTGGCACATTCCGGTTCAAATAAGCACAGAATTAATAATAGCCAATTCAAGAAGAATAAAGACAATAAACATGAGATGCCTGATGATGGGTTTGAGATAGCAGCCTGCTTTCTAACAACTGATCTCACAAAATACTGCTTAAATTGGAGGTACCAAGTTATCATCCCCTTTGCGCGTACATTGAACTCAATGTATGGTATACCCCATCTGTTCGAATGGATACATTTAAGGCTAATGCGAAGCACTCTCTATGTCGGTGATCCCTTCAATCCTCCATCAGATCCTACCCAACTTGACCTTGATACAGCTCTCAACGATGATATATTTATAGTTTCCCCTCGTGGAGGAATCGAGGGTTTATGTCAAAAATTATGGACTATGATTTCCATCTCAACAATTATATTATCCGCAACTGAGGCAAACACTAGAGTTATGAGCATGGTTCAGGGTGACAACCAAGCAATTGCAATCACCACTAGGGTAGTACGCTCGCTCAGTCATTCCGAGAAGAAGGAGCAAGCTTATAAAGCGAGTAAATTATTCTTTGAAAGGCTTAGAGCTAACAACCATGGAATTGGACACCACTTAAAAGAACAAGAAACAATCCTTAGTTCTGATTTCTTCATATATAGTAAGAGGGTGTTTTACAAAGGTCGAATTTTGACTCAGGCGTTAAAGAATGTGAGCAAGATGTGCTTAACAGCTGATATACTGGGGGACTGCTCACAAGCATCATGCTCCAATTTAGCCACTACTGTAATGCGCCTGACTGAGAATGGGGTCGAGAAAGATTTGTGTTATTTTCTAAATGCATTCATGACAATCAGACAATTATGTTATGATCTAGTATTTCCCCAAACTAAATCCCTTAGTCAGGACATCACTAATGCTTATCTCAATCATCCAATACTTATCTCAAGATTGTGTCTATTACCATCTCAATTGGGGGGACTGAACTTTCTCTCGTGTAGTCGCCTGTTCAATAGAAACATAGGTGACCCATTAGTGTCTGCAATTGCTGATGTGAAACGATTAATTAAAGCTGGCTGTTTAGACATCTGGGTCTTGTATAACATCCTTGGAAGAAGACCTGGGAAAGGTAAGTGGAGCACTCTGGCAGCTGATCCCTATACTTTAAACATAGATTATTTAGTTCCTTCAACAACTTTTTTAAAGAAGCATGCACAATATACATTGATGGAACGGAGTGTTAATCCCATGCTCCGTGGAGTATTCAGTGAAAATGCAGCTGAGGAAGAGGAGGAACTCGCACAGTATCTATTGGATCGTGAGGTAGTCATGCCCAGGGTTGCACATGTAATACTTGCCCAGTCTAGTTGCGGTAGAAGAAAGCAGATTCAAGGTTACTTGGATTCCACTAGGACTATTATCAGGTATTCATTGGAAGTGAGACCATTGTCAGCAAAGAAGCTGAATACAGTTATAGAATATAACTTATTGTATCTGTCCTATAATTTGGAGATTATTGAAAAACCCAATATAGTCCAGCCATTTTTGAATGCAATCAGTGTTGATACTTGTAGCATAGATATAGCTAGGTCCCTTAGAAAACTATCCTGGGCAACTTTACTTAATGGACGTCCCATCGAGGGATTAGAAACACCTGATCCCATTGAATTGGTACATGGGTGTCTGATAATTGGGTCAGATGAATGTGAGCATTGCAGTAGTGGTGATGACAAGTTTACCTGGTTTTTCCTACCTAAGGGGATAAGGCTAGATAATGACCCGGCATCCAACCCACCCATCAGAGTACCTTATATTGGATCTAAAACAGATGAGCGGAGGGTGGCGTCAATGGCTTACATCAAAGGAGCATCTGTATCACTTAAATCAGCACTCAGGTTAGCGGGGGTATATATTTGGGCTTTCGGAGATACAGAAGAATCATGGCAAGATGCCTATGAGTTAGCTTCCACTCGTGTTAATCTCACGCTAGAGCAATTGCAATCTCTCACTCCTTTGCCAACATCTGCCAACCTAGTCCACAGATTGGATGATGGTACTACTCAATTAAAATTTACCCCGGCAAGCTCTTATGCATTCTCTAGCTTTGTGCATATATCTAATGACTGTCAAGTTCTTGAGATCGATGATCAGGTAACAGATTCTAACCTGATTTACCAGCAAGTTATGATTACTGGCCTTGCTTTAATTGAGACATGGAACAATCCTCCAATTAACTTCTCCGTTTATGAAACTACACTACACCTTCACACAGGCTCATCTTGCTGTATAAGACCTGTTGAGTCTTGTGTAGTAAATCCTCCTTTGCTTCCTGTCCCCTTCATCAATGTTCCTCAAATGAATAAGTTTGTATATGACCCTGAACCACTCAGTTTGCTAGAGATGGAAAAAATTGAGGATATTGCTTATCAAACCAGAATTGGTGGTTTAGATCAAATCCCACTTCTGGAAAAAATACCCTTACTAGCTCACCTCACCGCCAAGCAGATGGTAAATAGCATCACTGGGCTTGATGAAGCAACATCTATAGTGAATGACGCTGTAGTTCAAGCAGACTATACTAGCAATTGGATTAGTGAATGCTGCTATACTTACATTGATTCTGTGTTTGTTTATTCTGGCTGGGCATTATTATTGGAACTGTCGTACCAAATGTACTACTTAAGAATTCAAGGCATCCAAGGAATTCTAGACTATGTGTATATGACCTTGCGGAGGATACCTGGAATGGCTATAACAGGCATCTCATCCACAATCAGTCACCCTCGTATACTCAGAAGATGCATCAATTTGGATGTCATAGCTCCAATCAATTCTCCACACATAGCTTCACTGGATTACACAAAATTAAGCATAGATGCAGTAATGTGGGGAACTAAGCAAGTTTTGACCAACATTTCGCAAGGTATCGATTATGAGATAGTCGTTCCTTCTGAAAGCCAGCTCACACTCAGTGATAGAGTTCTAAATCTAGTTGCTCGAAAATTATCACTACTTGCAATCATCTGGGCAAATTATAACTATCCTCCAAAGGTTAAAGGTATGTCACCTGAGGACAAATGTCAGGCTTTAACTACACACCTACTCCAGACTGTCGAGTATGTTGAGCACATTCAGATTGAAAAGACAAACATCAGGAGGATGATTATTGAACCGAAATTAACTGCCTACCCTAGTAATTTGTTTTATCTATCTCGAAAGCTGCTTAATGCTATTAGAGATTCTGAAGAAGGACAATTCCTGATTGCATCTTATTATAACAGTTTTGGATATTTGGAACCAATACTAATGGAATCTAAAATATTCAATCTAAGTTCATCTGAATCGGCATCTCTTACAGAGTTCGATTTCATCCTCAACTTGGAATTGTCTGAAACCAGCCTTGAGAAATACTCTCTCCCAAGTTTGCTTATGACTGCTGAGAACATGGATAACCCATTTCCTCAACCCCCCCTTCATCATGTTCTCAGACCATTAGGTTTATCATCCACCTCATGGTATAAAACAATCAGTGTTTTGAATTATATTAGCCATATGAAGATATCCGACGGTGCCCATCTATACTTGGCAGAGGGAAGTGGAGCCTCTATGTCACTTATAGAGACTTTCTTGCCCGGTGAAACAATATGGTACAACAGCCTATTCAATAGTGGTGAGAATCCTCCCCAACGCAATTTCGCCCCTTTGCCCACCCAATTTATTGAAAGTGTCCCTTACAGATTGATTCAAGCAGGTATAGCAGCAGGAAGTGGTGTAGTGCAAAGTTTCTATCCACTCTGGAACGGTAACAGCGATATCACTGACTTAAGCACGAAAACTAGTGTCGAATACATTATTCACAAGGTGGGAGCAGATACATGTGCATTGGTTCATGTGGATTTGGAGGGTGTACCCGGTTCAATGAATAGCATGCTGGAGAGAGCCCAAGTACATGCGCTACTGATCACAGTAACTGTATTAAAGCCAGGTGGCTTGCTAATCTTGAAAGCTTCATGGGAACCTTTTAATCGATTTTCCTCTTTACTCACAATCCTCTGGCAGTTCTTTTCGACAATAAGGATCCTGCGATCTTCATACTCTGACCCGAATAATCACGAGGTATACATAATAGCTACATTAGCAGTTGATCCCACCACATCCTCCTTTACAACTGCTCTGAATAGGGCGCGCACCCTAAATGAACAGGGCTTTTCACTCATTCCACCTGAATTAGTGAGTGAGTACTGGAGGAGGCGCGTTGAACAAGGGCAAATTATACAGGATCGTATAGATAAAGTCATATCAGAATGTGTCAGAGACCAATACCTGGCAGACAACAACATTATCCTTCAGGCAGGAGGGACTCCAAGCACAAGAAAATGGTTGGATCTACCTGACTATCCGTCGTTCAATGAATTACAATCGGAGATGGCCAGACTCATAACAATTCATCTCAAAGAGATAATAGAAATCCTAAAAGGCCAATCATCAGATCATGACACCTTATTATTTACTTCGTACAATGTAGGTCCCCTCGGGAAAATAAATACAATACTCAGATTGATTGTCGAGAGAATTCTTATGTACACTGTAAGGAACTGGTGCATCTTGCCCACCCAAACTCGTCTCACCTTACGACAGTCTATCGAGCTTGGAGAGTTTAGACTAAGAGACGTGATAACACCCATGGAGATCCTTAAGCTATCCCCCAACAGGAAATATCTGAAGTCTGCATTAAACCAATCAACATTCAATCATCTAATGGGGGAAACATCTGACATATTGTTAAATCGAGCCTATCAGAAGAGAATTTGGAAAGCCATTGGATGTGTAATCTATTGCTTTGGTTTGCTTACCCCGGATATTGAAGATTCCGAGCGCATTGATATTGATAATGATATACCTGATTATGATATCCACGGGGACATAATTTAAATCTGTTAAAGACTCCTCTGGTATGATACATCACCAAAAGGTGCCACACCAGCATCCCAATTCTTCTAGACCGCACACGACCTCGAACAATCATAACCACATCAGTATTAAATCCAGAAGATCCTTTTAAGAAAAAATTGATTCTACTTTCTCCCCTTGGT
